# Strategies for Effective Communication in Hypertension Management: Validation of Messages from a Mobile Application to Assist Hypertensive Older Adults in Adherence to Treatment, Nutrition and Physical Activity

**DOI:** 10.3390/nu16244284

**Published:** 2024-12-12

**Authors:** Alayne Pereira, Raiza Trombini, Yuri Barbalho, Marina Stival, Luciano Lima, Renata Zandonadi, Verônica Ginani, Rafaella Dusi, Silvana Schwerz Funghetto

**Affiliations:** 1 Graduate Program in Health Sciences and Technologies, University of Brasília, Campus Universitario Ceilândia, Brasília 72220-275, Brazil; raiza.lima@aluno.unb.br (R.T.); yurigustavo.sousa@gmail.com (Y.B.); marinamorato@unb.br (M.S.); ramosll@unb.br (L.L.) silvanasf@unb.br (S.S.F.); 2 Department of Nutrition, Faculty of Health Sciences, University of Brasília, Campus Universitario Darcy Ribeiro, Brasília 70910-900, Brazil; renatapz@unb.br (R.Z.); vcginani@unb.br (V.G.); rafaella.dusi@aluno.unb.br (R.D.)

**Keywords:** hypertension, mHealth, preventive medicine, prehypertension, text messages, validation studies, nutrition, mobile health interventions

## Abstract

Background: Poor adherence to antihypertensive treatment is a common problem among elderly hypertensive patients and one of the leading causes of inadequate blood pressure control. In this sense, it is essential to improve strategies for effective communication in managing hypertension treatment for this group. Objective: This study aimed to validate the text messages of a mobile application to aid adherence to antihypertensive treatment, nutrition, and physical activity among older adults with hypertension treated in Brazilian public primary health care. Methods: This descriptive, methodological development study with a quantitative approach was carried out between March and August 2024. Results: A total of 27 messages were constructed and validated by 13 experts, and this stage was divided into two rounds. The Content Validity index and percentage of agreement were used in the validation process. The messages were developed using theory, national guidelines, validation, and expert review. Conclusions: Text messages for adherence to antihypertensive treatment involving medication, nutrition, and physical activity have enormous potential with the target audience studied.

## 1. Introduction

Population aging, reinforced by changes resulting from the demographic and epidemiological transition, has led to a significant increase in the prevalence of chronic non-communicable diseases (NCDs) [1,2]. NCDs are the leading cause of morbidity and mortality in the general population and, among older adults, they are the leading cause of dysfunction in most South American countries, including Brazil [3].

Aging is part of life, and its protection is a social right, Brazil has a law that states that it is the obligation of the family, the community, society, and the public authorities to ensure that the elderly enjoy the right to life, health, food, education, culture, sport, leisure, work, citizenship, freedom, dignity, respect, and family and community life. According to this law, a citizen aged 60 or over is considered an elderly person [4]. Given this scenario, it becomes essential to develop public policies that address the needs” of this aging population.

From another perspective, aging is recognized as progress in society, reflecting social, technological, and health advances. Adopting healthy habits can help reduce the risk of developing NCDs, favoring weight control, improving quality of life, and strengthening mental health. It is essential to emphasize the importance of continuous monitoring, and the sustainability of interventions aimed at promoting health [5].

The report “Leaving No One Behind in an Ageing World” predicts that the global older adult population will reach 1.6 billion by 2050 [6]. In Brazil, until the 1970s, families were large and rural and had high infant mortality rates; however, this reality has changed [7]. Projections for 2050 indicate that Brazil will have the sixth-largest older adult population in the world, with around 32 million people, representing 16% of the total population [8].

Hypertension (HT) is the most prevalent chronic non-communicable disease (NCD) in the Brazilian population. It is defined by blood pressure levels at which the benefits of treatment, whether pharmacological or not, outweigh the risks. The primary objective of antihypertensive treatment, including pharmacological and non-pharmacological measures, is to reduce the morbidity and mortality associated with high blood pressure [9].

Poor adherence to pharmacological treatment is common among older adults and it is one of the leading causes of inadequate blood pressure control [10]. Whether intentional or not, poor adherence to treatment is the primary reason for therapeutic failure in hypertension. Related factors include physical, mental, and psychological changes resulting from aging, in addition to a lack of knowledge about hypertension and its impacts on health [11].

Management of hypertension also includes non-pharmacological methods, such as dietary changes and physical exercise. However, hypertension control remains inadequate, even in developed countries, where only 29% to 50% of treated patients achieve blood pressure control [12,13].

New patient-centered initiatives are essential for improving adherence to pharmacological and non-pharmacological measures. Evidence suggests that patient self-management of chronic disease can increase motivation, understanding, skills, quality of life, and clinical outcomes, in addition to promoting efficient resource use [14].

Digital tools, such as mobile applications, can support initiatives aimed at reducing hypertension by enhancing communication and facilitating lifestyle modification behaviors. Mobile health technologies have shown remarkable effectiveness in disease prevention and control, especially as part of non-pharmacological treatment for older adults with chronic diseases, facilitating treatment adherence and self-monitoring [15].

Older adults have been shown to respond well to instant messaging applications, and exposure to educational messages can improve the habits of patients with chronic diseases, even in less developed areas. A clinical trial in the United Kingdom showed that patients with type 2 diabetes or hypertension who received personalized text messages for 12 weeks significantly improved their adherence to medication treatment. Similar results were observed in studies with African Americans, where text messages were also effective as medication reminders [16]. Therefore, patient education and behavioral therapy measures are recommended to promote adherence to antihypertensive treatment. This study aims to validate text messages from a mobile application to assist in treatment adherence in older adults with arterial hypertension treated in primary care.

## 2. Materials and Methods

### 2.1. Study Design

**Figure 1:**
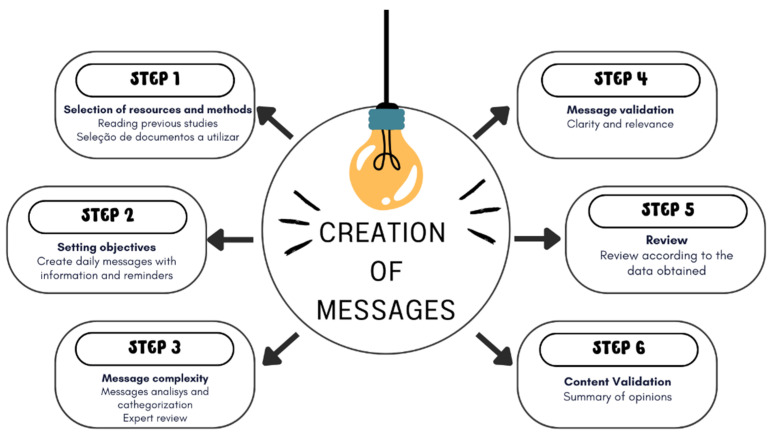
Steps used to construct the messages.

This is a descriptive, methodological development study with a quantitative approach carried out between March and August 2024. The method developed by Goes was adapted to create the messages, which present six steps described in Figure 1.

### 2.2. Messages Construction

In step 1, Selection of resources and methods, previous studies that addressed the topic and used the practice of sending messages to older adults with Arterial Hypertension were read. The Brazilian Guideline for Arterial Hypertension was used to create messages for the target audience.

In step 2, Establishing objectives, the objective was established to create daily messages with important information about adherence to medication treatment and reminders so that the user does not forget to take their daily medications.

In step 3, Message complexity, messages were developed and categorized as reminders (8 messages), knowledge (7 messages), motivational (5 messages), and behavioral (12 messages). Undergraduate and graduate faculty in nursing, nutrition, health literacy, and pharmacy were invited to review the messages.

In Step 4, Message validation, assessed the clarity and relevance of the messages were assessed.

In step 5, Guidance, the messages were reviewed according to the results obtained from data analysis and processing.

In step 6, Content validity and making changes, the material was made available to the experts in the second round after the consolidation of the first validation stage, thus obtaining a summary of the opinions and suggestions sent, and the arguments of the team responsible for the questionnaire.

### 2.3. Sampling Calculation

The selection of experts was carried out by convenience and intentional sampling, with the following inclusion criteria: having a master’s or PhD degree in the health area; having published work on mHealth/Hypertension/Medication Adherence; having a clinical practice of at least one year in Primary Health Care.

### 2.4. Message Validation

The Delphi method was used to validate the proposed messages. This method allows to gather opinions from geographically distant experts, generating in-depth results on complex topics. Potential experts were identified on the Lattes Platform of the National Council for Scientific and Technological Development, resulting in 61 candidates who met the inclusion criteria.

The experts were selected using the method proposed by Fehring [17], with adaptations. The inclusion criteria for this group to participate in the study were: being a health professional, achieving a score > 5 according to Table 1, voluntarily accepting to participate in the research, and responding to the entire proposed form.

After being invited by email, the experts who expressed an interest in participating in the message validation process received the informed consent form, the expert characterization form and the messages to be validated by the same means. A deadline of 15 days was set for submitting the evaluation.

The evaluation instrument included messages to be analyzed and options based on two criteria (clarity and criterion). The first is clarity, which indicates whether the information presented is easy to understand and whether it is clear in conveying the content covered in the messages: Yes or No. The second criterion, degree of relevance, assessed the degree of importance, taking into account the impact, motivation, and interest attributed to the message, measured using a Likert scale with increasing values from 1 to 4: Irrelevant, Slightly relevant, Relevant, and Very relevant. The instrument also included space for additional considerations and suggestions.

The first round’s consolidation consisted of synthesizing the opinions and suggestions sent and the arguments of the questionnaire team. In cases where the minimum agreement rate and/or the content validity index were not reached, the revised material was then made available to the experts in a second round.

### 2.5. Data Analysis and Processing

The information from the assessment instrument was organized in Excel for Windows and analyzed using the Content Validity Index (CVI), which measures the proportion of experts who agree on evaluating the messages. The CVI was calculated using two mathematical equations: S-CVI/Ave (average of the content validation indexes for all messages) and I-CVI (content validity of individual items). A CVI of 0.90 was established as the standard to establish the excellence of the content validity of the messages.

The percentage of general agreement among the experts regarding the clarity of the messages was assessed using the formula: number of items with responses divided by the total number of items assessed, multiplying the result by 100. A minimum agreement rate of 75% was assigned.

### 2.6. Ethical Considerations

The study protocol, field instruments, and informed consent forms were reviewed and, approved by the Research Ethics Committees of the Faculty of Ceilândia of the University of Brasília—CEP/FCE (5.637.553 and CAAE: 62700422.0.0000.8093). All participants were informed about the study and gave oral consent.

## 3. Results

In the message selection and construction stage, Brazilian literature and guidelines were consulted. The aspects addressed in the literature and the Brazilian Guideline, considered essential to support treatment adherence, were used to select and construct the messages, and the messages were categorized. The main results of the categorization determined the messages to be used and sent to the specialists. The messages categorized, reviewed, and validated in the first and second rounds are presented in Table 2.

After creating the messages, they were sent to the expert committee for evaluation. Eight of the sixty-one experts invited refused to participate. Therefore, the messages were sent to 53 experts who met the inclusion criteria and agreed to participate. However, only 13 answered the first round of evaluation (response rate of 24.5%). Most have an average age between 31 and 39 years (53.8%) and a predominance of females (92.3%). Regarding training, professionals participated in medicine (7.7%), pharmacy (23.1%), nursing (53.8%), and other areas (14.1%), including specialists in health literacy.

**Figure 2:**
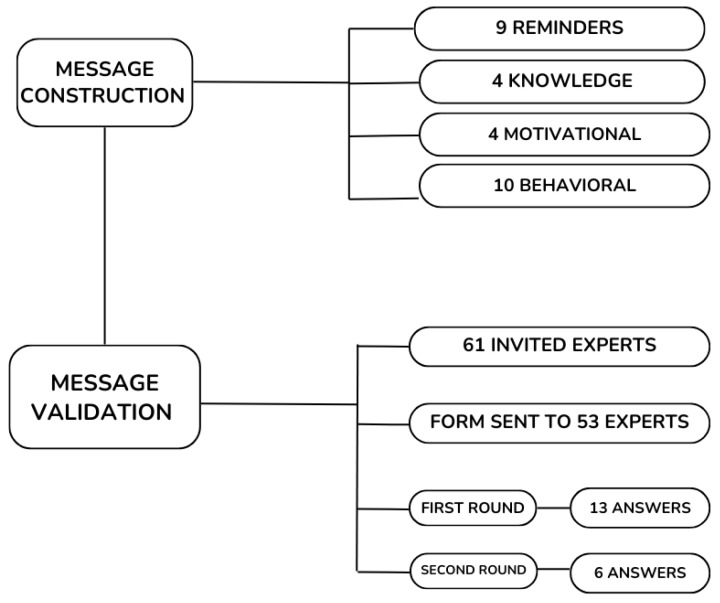
Message construction and validation diagram.

Most specialists have between 10 and 15 years of professional experience (38.5%), a master’s degree in Chronic Diseases and/or educational technologies (84.6%), and work in research (92.3%). In the second round, six experts participated (a response rate of 46.1% compared to the first round) (Figure 2).

Regarding the experts’ evaluation process on messages’ clarity, the knowledge and motivational categories presented a lower percentage of agreement among the experts (61.5% and 61.6%) in round 1 (Table 3). Regarding the general agreement of the experts about messages’ clarity after they were reviewed and evaluated in round 2, 91.8% considered the information in the messages to be clear. The I-CVI evaluation of each category was also above 0.90 in all categories, and the general assessment was 1.0 after reviewing the messages (Table 3).

## 4. Discussion

This study describes methodological steps to validate the text messages sent in an app to help older adults improve adherence to hypertension treatment. The messages were validated based on the high CVI and the experts’ agreement percentage.

Like other low- and middle-income countries, Brazil has experienced a significant technological transition in recent years. Text messaging has become the primary form of technology-mediated communication and the most common activity on mobile phones. However, despite its benefits, text messaging is not without its risks [15]. The validation process concerns making something valid, that is, content validation makes it possible to assign value to a construct [18].

Different technologies have been used to deliver support messages, reminders, and information to patients, and as a result, most studies and reviews point to the importance of adapting the content of messages to the target audience. Regarding content validation by experts, the selection of specialists from different contexts allows the technology to be adapted to reach the target audience [19]. The CVI values and percentage of agreement were satisfactory, ensuring the validity and reliability of the messages developed. The CVI values and percentage of agreement were satisfactory, ensuring the validity and reliability of the developed messages.

Medication nonadherence is a leading cause of uncontrolled hypertension. Only 50% of patients adhere to chronic treatments correctly, and poor adherence is considered a “silent epidemic”, contributing to 21–37% of preventable adverse drug events. As a result, there is increased morbidity and mortality, as well as significant additional costs to health care systems. This problem is particularly prevalent among older adults, accounting for up to 10% of hospitalizations. In the United States, it is estimated that the health care system costs between $100 and $300 billion (or more) annually [20].

Patients are known to do most of the self-management of hypertension. A systematic review demonstrated that medication adherence and self-management behavior showed positive changes after mobile health (mHealth)-based self-management. Methods included patient education, self-monitoring of clinical data and behavior, self-titration of medical management, and support for medication adherence according to prescriptions.

The Brazilian Guideline for Arterial Hypertension introduced a specific chapter on adherence to antihypertensive treatment and discussed aspects and strategies for improving therapeutic adherence. It highlights the need to increase the skills of people with hypertension, mainly related to reminders, knowledge, and motivational messages that encourage behavioral changes [21].

Text messages serve as reminders to reinforce daily medication intake and address the issue of forgetfulness, which has been identified as one of the barriers to medication adherence. The WHO multidimensional adherence model [22], in which it is necessary to offer advice, support, and information so that patients can understand the importance of maintaining blood pressure control throughout the day, using their medications rationally, learning how to deal with missed doses, and identifying adverse events and what to do when they occur.

In this study, we validated seven messages related to reminders. Studies have shown that sending messages as reminders increases adherence to treatment for patients with chronic diseases, constituting an important tool for promoting self-care.

Several strategies have been used, including sending text messages as reminders. Messages developed involving medication topics provided information on how medications worked, typical side effects, and tips on taking medications regularly, as well as nutrition, self-care, motivation, and support [23,24,25].

The use of simple text message reminders for blood pressure (BP) monitoring may increase patient adherence to their daily antihypertensive medication regimen. Thus, increased adherence may improve their BP and potentially decrease cardiovascular disease morbidity and mortality [26].

In this context, technology is an important ally, as messages offer an innovative opportunity to improve treatment adherence, which is extremely useful in the treatment of hypertension. Greater self-efficacy is associated with better initiation and engagement in self-care behaviors, such as medication adherence, physical activity, and dietary changes, among patients with hypertension [27].

The benefits of text messaging programs to promote lifestyle changes have been increasingly recognized. These programs, which are based on principles of behavior change and provide self-care advice through a widely accepted form of communication (text messaging), effectively promote lifestyle changes [28].

Studies show that mobile applications are practical, feasible, and accepted by older people, leading to improvements in well-being, stress reduction, and a decrease in depressive symptoms. Mobile apps and text messaging have been shown to be effective in significantly reducing anxiety, stress, and depression among users. Therefore, mobile interventions, including text messaging, are effective in increasing physical activity and reducing sedentary behavior in adults aged 50 and over [29,30,31].

Two studies delivered interventions exclusively through educational and motivational text messages on mobile phones about hypertension and its medical therapy [28,29], while another study used only a mobile app. This app allowed participants to record personal data, set recommended blood pressure target levels, record medical advice about prescribed treatment, set reminder alarms, schedule appointments or events, and record blood pressure measurement results [32].

The use of messages with positive and negative framing is highlighted, with the application of diverse persuasive approaches to influence attitudes, beliefs, and intentions regarding adopting and promoting healthy behaviors. Negative messages are not intended to punish but rather to raise awareness of the undesirable complications associated with a lack of adherence to drug treatment, thus reinforcing the importance of following therapeutic recommendations [33].

Interventions that address multiple factors, such as education and behavior or habits, are more likely to be successful than those that focus on just one aspect [34]. Hypertensive patients require self-management and motivation, in addition to pharmacotherapy, for adequate disease management and BP control. Text messages can also be used to educate patients and improve communication between the patient and the health care team in a cost-effective manner. They can be incorporated as a daily routine tool to enhance the management of serious diseases [35].

The patient experience of using technologies to support self-monitoring of BP generates empowerment, characterized by an improved perception and understanding of hypertension, a sense of control over one’s own health, safety, and greater patient responsibility [36].

Through self-management education, older adults can learn strategies to manage their chronic condition and live a healthier life. Therefore, educational interventions have significant roles in the effective management of hypertension. Patient education improves patients’ knowledge about the disease and its complications and treatments, helps them modify their lifestyles, and promotes their adherence to medication [37].

Digital behavioral health interventions have reduced BP, and increased patient knowledge, and adherence to healthy behaviors. Several of these behaviors interact dynamically and influence peers, leading to regular BP monitoring, so technologies can help individuals better manage their condition and ultimately improve BP control [38].

However, it is observed that the improvement in medication adherence is intrinsically related to the reduction in emergency room visits and, consequently, to a lower number of hospitalizations and mortality rates among older adults with diabetes and hypertension [39].

The messages were designed for older adults and used accessible, objective, and precise language. E-Health literacy was positively associated with positive outcomes in health promotion behaviors, self-care, medication adherence, health knowledge and attitudes, and health decision-making. Literacy skills incorporate someone’s ability to seek, find, understand, and evaluate online health information and apply the acquired knowledge to address health problems [40].

Messages must undergo a validation process by experts in the field to be considered appropriate and credible for sending to the target audience. In developing these messages, it is essential to involve a multidisciplinary team with experts in technology, health, and representatives of the target audience. Validating the content in the initial phases of the study is crucial to ensure that the messages accurately fulfill their purpose [41,42,43].

### Limitations

There are some limitations to this study. Firstly, the Delphi technique does not allow for face-to-face or group interaction between participants, which restricts the direct exchange of information. However, it favors greater freedom and autonomy in the evaluations. In addition, the prolonged duration of the validation process, which can take several months before all the responses are obtained, presents a challenge. It is worth noting that this study focuses on content validation and experts’ percentage of agreement with the instrument, while future research should investigate its psychometric properties and dimensionality.

## 5. Conclusions

The experts involved must know about the topic to assess the relevance and clarity of the content. Thus, improvements were made to the messages based on the experts’ comments and suggestions. The messages developed in this study were designed for the cultural context of older adults in Brazil, and cultural adaptation is recommended so that they can be used in other countries.

This study reports the development and validation of 27 mobile phone text messages that are now suitable for clinical use. The messages were developed using theory, national guidelines, validation, and expert review. Text messages for adherence to antihypertensive treatment have enormous potential with the target audience studied. We report a development process to ensure the messages are evidence-based, appropriate, and useful.

## Figures and Tables

**Table 1:** Scoring system and criteria adapted from Fehring for selecting experts.

Criterion	Scoring
Holds a master’s/PhD in the health field	3 points
Master’s degree with dissertation on Hypertensive older adults/mHealth/Medication adherence	2 points
PhD in the area of Hypertensive older adults/mHealth/Medication Adherence	2 points
Has published studies on Hypertension/mHealth/Medication Adherence/Primary Health Care	2 points
Has published studies on health education/validation studies	2 points
Has recent clinical practice of at least one year in Primary Health Care	1 point
Has training (specialization) in hypertense older adults/mHealth/Medication Adherence	2 points
Total	14 Points

**Table 2:** Messages sent to experts for evaluation.

Category	First Round–Messages in Portuguese	First Round–Free Translation of the Messages to English	Second Round–Messages in Portuguese	Second Round–Free Translation of the Messages to English
Reminder	Lembre-se de tomar todos os seus medicamentos seguindo dose e horário prescritos. Não deixe de tomar nenhum comprimido!	Remember to take all your medications according to the prescribed dose and schedule. Don’t miss any pills!	Lembre-se de tomar os seus medicamentos no horário correto, conforme o médico prescreveu	Remember to take your medication at the correct time, as your doctor prescribes.
	Se você tem medicação prescrita à noite, lembre-se de tomá-la!	If you have medication prescribed for the night, remember to take it!	Se você tem algum comprimido para tomar à noite, lembre-se de tomá-lo!	If you have any pills to take at night, remember to take them!
	Planeje-se! Se os seus comprimidos para este mês estiverem acabando encaminhe-se ao Centro de Saúde para solicitar o medicamento em tempo hábil.	Plan ahead! If you are running out of pills for this month, go to the Health Center to request the medication in good time.	No modifications	
	Siga corretamente os horários e doses indicadas pelo seu médico.	Follow the times and doses indicated by your doctor correctly.	No modifications	
	Uma forma bacana de lembrar de tomar: deixe-o perto da escova de dentes ou da xícara de café da manhã.	A great way to remember to take it: leave it near your toothbrush or morning coffee cup.	Uma forma bacana de você lembrar de tomar seus medicamentos, é colocá-los perto xícara de café da manhã.	A great way to remember to take your medications is to place them near your morning cup of coffee.
	Se você precisar tomar medicação quando estiver fora de casa, programe o alarme do seu celular para lembrá-lo.	If you need to take medication while you are away from home, set an alarm on your cell phone to remind you.	No modifications	
	Lembre-se de tomar seus medicamentos para controlar sua pressão. Queremos cuidar de você!	Remember to take your medication to control your blood pressure. We want to take care of you!	Lembre-se de tomar seus medicamentos para controlar sua pressão. Queremos ajudar a cuidar de você!	Remember to take your medication to control your blood pressure. We want to help take care of you!
Knowledge	Os medicamentos para pressão arterial atuam de diferentes formas. Se você suspender um comprimido ele não fará efeito.	Blood pressure medications work in different ways. If you stop taking a pill, it will not be effective.	Os medicamentos para o tratamento da hipertensão possuem diferentes formas de agir no organismo. Se você deixar de tomar algum comprimido, o tratamento não fará efeito.	Medications for treating hypertension have different ways of acting on the body. If you miss a pill, the treatment will not be effective.
	Caso sinta algum desconforto relacionado a sua medicação, por favor nos informes.	Please let us know if you experience any discomfort related to your medication.	Caso não se sinta bem ao tomar os seus medicamentos, procure o médico ou uma unidade de saúde.	If you do not feel well while taking your medication, seek medical advice or a health unit.
	Procure espaço e momentos de tranquilidade para relaxar, o estresse afeta a sua pressão.	Look for space and moments of tranquility to relax, stress affects your blood pressure.	No modifications	
	Caso esqueça de tomar o medicamento de manhã, tome assim que possível.	If you forget to take your medicine in the morning, take it immediately.	No modifications	
	A hipertensão é definida como a pressão arterial sistólica ≥ 140 mmHg ou pressão arterial diastólica ≥ 90 mmHg.	Hypertension is defined as systolic blood pressure ≥140 mmHg or diastolic blood pressure ≥90 mmHg.	A hipertensão é definida como a pressão arterial sistólica maior que 140 mmHg ou pressão arterial diastólica maior que 90 mmHg.	Hypertension is defined as systolic blood pressure greater than 140 mmHg or diastolic blood pressure greater than 90 mmHg.
	Estar sempre com a pressão arterial alta pode causar doenças cardiovasculares, acidente vascular cerebral e doenças renais.	Having high blood pressure all the time can cause cardiovascular disease, stroke and kidney disease.	Estar sempre com a pressão alta pode causar doenças no coração, derrame (AVC) e doença nos rins.	Having high blood pressure all the time can cause heart disease, stroke, and kidney disease.
Motivational	Que bom que você está mantendo a sua pressão normal. Continue a tomar o medicamento conforme prescrito.	It’s good that you are keeping your blood pressure normal. Continue to take your medication as prescribed.	No modifications	
	Ao controlar a pressão arterial, você adiciona anos à sua vida. Lembre-se de tomar seus medicamentos para controlá-lo. Queremos cuidar de você!	By controlling your blood pressure, you can add years to your life. Remember to take your medications to control it. We want to take care of you!	Ao controlar a pressão arterial, você adiciona anos à sua vida. Mantenha o tratamento. E conte conosco. Estamos aqui para te ajudar!	By controlling your blood pressure, you can add years to your life. Keep up with your treatment. And count on us. We are here to help you!
	Se precisar de ajuda, não tenha medo de perguntar!	If you need help, don’t be afraid to ask!	Se precisar de ajuda, não tenha vergonha ou medo de perguntar!	If you need help, don’t be shy or afraid to ask!
	Você já aferiu a pressão? Faça a aferição de sua pressão arterial semanalmente.	Have you ever checked your blood pressure? Check your blood pressure weekly.	Você já mediu sua pressão arterial? Faça a medida de sua pressão arterial toda semana.	Have you ever measured your blood pressure? Measure your blood pressure every week.
Behavioral	Cuidado com o sal na alimentação, em excesso pode aumentar a sua pressão arterial!	Be careful with salt in your diet, too much can increase your blood pressure!	Lembre-se de prestar atenção na quantidade de sal que você coloca na comida, uma quantidade grande de sal pode aumentar sua pressão.	Remember to pay attention to the amount of salt you put in your food, a large amount of salt can increase your blood pressure.
	Cuidado com a ingestão produtos que contenham muito sal, como salsichas, enlatados e sopas instantâneas!	Be careful when eating products that contain a lot of salt, such as sausages, canned foods and instant soups!	Procure evitar comer produtos que contém muito sal, como salsichas, presunto, mortadela, conservas e sopas instantâneas!	Avoid eating products that contain a lot of salt, such as sausages, ham, mortadella, preserves and instant soups!
	Já experimentou diminuir a quantidade de sal da sua alimentação? Tente tirar o saleiro da mesa. Isso já é uma melhoria.	Have you tried reducing the amount of salt in your diet? Try removing the salt shaker from the table. That will already be an improvement.	No modifications	
	Diminua o excesso de óleo ao cozinhar: experimente cozinhar no vapor, assar ou fazer churrasco	Reduce excess oil when cooking: try steaming, baking or barbecuing	Diminua o excesso de óleo ao cozinhar: experimente cozinhar no vapor, assar ou grelhar os alimentos.	Reduce excess oil when cooking: try steaming, baking or grilling your food.
	Evite fumar.	Avoid smoking.	No modifications	
	Priorize em sua alimentação carnes de aves sem pele e sem gordura.	Prioritize skinless and fat-free poultry in your diet.	Priorize sua alimentação! Sempre que possível, consuma carnes de aves sem pele e sem gordura.	Prioritize your diet! Whenever possible, consume skinless and fat-free poultry.
	Para tornar suas refeições mais atrativas, experimente adicionar uma nova fruta, vegetal ou erva.	Try adding a new fruit, vegetable or herb to make your meals more appealing.	Para tornar suas refeições mais atrativas e gostosas, experimente adicionar novas frutas, vegetais e ervas.	Try adding new fruits, vegetables and herbs to make your meals more appealing and delicious.
	Experimente cozinhar no vapor ou assar os alimentos para reduzir a necessidade de excesso de óleo ao cozinhar.	Try steaming or baking your food to reduce the need for excess oil when cooking.	No modifications	
	Exercite-se! O exercício auxilia a manter a normalidade de sua pressão arterial!	Exercise! Exercise helps keep your blood pressure normal!	No modifications	
	Comece a fazer atividade física duas vezes por semana e aumente gradativamente o número de dias.	Start physical activity twice a week and gradually increase the number of days.	No modifications	

**Table 3:** Judges’ assessment of the percentage of agreement and the CVI for each category, Brasília, Federal District, Brazil, 2024.

Category	Round 1		Round 2	
	AP	I-CVI	AP	I-CVI
Reminder	81.1	0.82	97.8	1.00
Knowledge	61.5	0.83	75.2	0.94
Motivational	61.6	0.76	95.8	0.96
Behavioral	80.8	0.80	98.3	0.92
Total S-CVI/Ave	71.2	0.8	91.8	1.0

I-CVI—Item Level Content validity index, AP—Agreement Percentage.

## Data Availability

The raw data supporting the conclusions of this article will be made available by the authors on request.
